# Examining the association between cultural self-construal and dream structures in China, Japan, and the United States

**DOI:** 10.3389/fpsyg.2025.1688407

**Published:** 2026-01-12

**Authors:** Hisae Konakawa, Toshio Kawai, Yasuhiro Tanaka, Chihiro Hatanaka, Qi Li, Alethea Koh

**Affiliations:** 1 Uehiro Research Division, Institute for the Future of Human Society, Kyoto University, Kyoto, Japan; 2 Kokoro Research Institute Kyoto, Kyoto, Japan; 3 Graduate School of Education, Kyoto University, Kyoto, Japan; 4 Institute for the Future of Human Society, Kyoto University, Kyoto, Japan

**Keywords:** culture, self-construal, independence-interdependence, dreams, empirical dream research, analytic and holistic thinking

## Abstract

**Introduction:**

Cultural differences in human relationships and values between Western and East Asian societies have been investigated, particularly in relation to independent versus interdependent self-construal. Our previous research demonstrated clear distinctions between American and Japanese dream structures: American dreams featured strong dream-ego agency, clear mobility, and definitive endings, whereas Japanese dreams were characterized by weaker agency, vagueness of the dream-ego, and greater presence of others in the dream narrative. While much attention has been given to self-construal differences between Western and East Asian societies, recognizing intra-East Asian heterogeneity is critical, as meaningful variations exist within this region. Building on this perspective, the present study highlights cultural differences within East Asia-particularly in dream structure and self-construal-while situating them in relation to broader East–West comparisons.

**Methods:**

This study utilizes previously collected data from Japan and the United States, and adds newly collected data from China to investigate and compare anthropophobia mentality, self-construal, and dream structure. A total of 250 participants were recruited from China via online platforms, and valid responses (*n* = 173) were statistically compared with archival data from the United States (*n* = 220) and Japan (*n* = 257).

**Results:**

Results revealed culturally distinct patterns in the relationships among self-construal and anthropophobia. In the Chinese sample, independent and interdependent self-construals were positively correlated, suggesting that these orientations may coexist rather than oppose each other. In contrast, they were negatively correlated in the Japanese and American samples. Moreover, in Japan and the United States, higher independent self-construal was associated with lower anthropophobia, whereas higher interdependent self-construal was associated with higher anthropophobia. No significant associations were observed in the Chinese sample, indicating that self-construal may not directly predict anthropophobia in this cultural context. Significant differences in dream patterns were also found between Americans and East Asians. Importantly, both similarities and differences between Chinese and Japanese dreams emerged, indicating that even within East Asia, distinct cultural traits related to ego orientation and conceptions of happiness may be reflected in dream themes.

**Discussion:**

These findings underscore the importance of examining intra-East Asian variation, offering new insights into how self-construal and cultural values shape the structure of dreams.

## Introduction

Previous studies have identified cultural differences in human relationships and values between Western and East Asian societies (for a review, see Markus and Kitayama, 1991). These cultural distinctions manifest at both conscious and unconscious levels as the human psyche is deeply embedded and influenced by the social environment.

One such cultural difference is found in self-construal, which refers to individuals’ own conceptualization of who they are, or how they experience themselves to be (Kitayama, 1994; Han and Humphreys, 2016). Many psychological processes such as thinking styles, emotion and motivation change in response to one’s self-construal (Cross et al., 2002; Shao et al., 2018). Particularly, self-construal described as independent or interdependent views of self, are commonly studied in comparisons between the West and East as representatives of each type of culture. Aspects of one’s culture, such as manners, language, customs, norms and social systems, foster culturally-specific self-construal due to the values and priorities made salient to the individual, which may unconsciously shape individuals’ perception and definitions of their selves. In the West such as the US, the independent self is defined by internal attributes which is less influenced by others and their surrounding environment. Whereas in East Asia such as China and Japan, the interdependent self is defined in relation to others and their surrounding environment, where the context exerts dominant influence (Markus and Kitayama, 1991). These cultures also show general differences in cognitive set or style between analytic thinking and holistic thinking (Nisbett et al., 2001).

Comparative studies between the United States and China have revealed notable cultural differences in self-perception grounded in distinct self-construal, particularly influenced by social contexts and interpersonal values (Liew et al., 2011; Dong et al., 2021). For instance, differences have been observed in the cognitive processing of self-related information between Americans and Chinese individuals. Findings suggest that, in social contexts, Chinese individuals are more susceptible to external influences, such as the perceived authority or status of others. For example, for Chinese participants the presence of a higher-status individual (e.g., a boss) can diminish the self-face recognition advantage typically observed in cognitive processing (Liew et al., 2011). Furthermore, studies on individual selfish behaviors have demonstrated that Americans tend to evaluate their own selfish actions as more acceptable than the same actions performed by others (Dong et al., 2021). In contrast, Chinese individuals exhibit a greater tendency toward self-sacrifice, which has been shown to positively affect both their own and others’ emotional well-being (Zhu et al., 2020). These inclination among Chinese individuals are believed to be strongly influenced by the interdependent self-concept, which emphasizes emotions and desires centered around others and the environment (Markus and Kitayama, 1991).

Confucian values continue to influence the cultural and spiritual framework of China, particularly through their role in fostering holistic thinking and the interdependent self-concept (Wang et al., 2022). At the same time, recent studies have noted a growing emphasis on the independent self-construal in contemporary Chinese university culture. For instance, possessing an independent self-construal has been linked to improved daily adjustment among university students (Lu et al., 2025). However, research examining broader cultural shifts in China has found that collectivism remains consistently associated with various life domains—particularly the workplace—and that there is no clear evidence of an increasing trend toward individualism over time (Hamamura et al., 2021). These findings suggest that both the traditional interdependent self-concept rooted in Confucianism and the emerging independent self-concept are simultaneously emphasized in modern Chinese society.

On the other hand, even among East Asian countries influenced by Confucian culture, which emphasizes interpersonal harmony, variations in cultural mentality have been suggested (Zhang et al., 2005). In both China and Japan, the contextual positioning of the self reflects culturally specific patterns, resulting in differences in the scope and nature of moral responsibilities within interpersonal relationships (Takamatsu et al., 2024). A comparative study between the United States, China, and Japan have revealed pronounced differences in emotional expression within social contexts (Ip et al., 2021), particularly between Western (American) and East Asian (Chinese and Japanese) societies, reflecting the contrast between independent and interdependent self-construal. However, subtle cultural variations have also been observed between Chinese and Japanese individuals, with Japanese individuals tending to rely more heavily on contextual cues. These findings underscore the importance of comparing East Asian countries to better understand the distinctive cultural mentalities underlying each society. Accordingly, this study compares the United States, China, and Japan to examine cultural differences in self-construal.

### Dream structures as reflections of self-construal

Building on our previous study (Konakawa et al., 2023), we note that cultural self-construal affects “thought processes,” “self-efficacy,” and “situations that make individuals feel happy” even at the unconscious level (Markus and Kitayama, 1991; McLaren et al., 2024; Suryaningrum, 2018; Uchida and Ogihara, 2012). Previous research in cultural products has shown that items produced by individuals (etc., stories, folklore, music) are influenced and shaped by the culture in which they are embedded (Kawai, 1995). In a similar manner as the cultural narratives, dream content is also embedded within cultural contexts. For instance, analytical psychology assumes that “dreams contribute to the self-regulation of the psyche by automatically bringing up everything that repressed or neglected or unknown” (Jung, 1952). The self in dreams is referred to as the dream-ego, which has been regarded as “the potential image of the ego” (Kawai, 1971). The dream-ego is differentiated from one’s actual self, being more like an actor representing the self within a dream. Given that the dream-ego has the agency to think within the dream, the dreamer’s values, beliefs and cultural background could also shape how the dream-ego acts in the dream. Therefore, the actions and thoughts of the dream-ego in the dream may reflect dreamer’s self-construal, as in real life. This perspective corroborates with recent brain injury research from the field of neuroscience, where a connection between the state of dreaming and the default mode network of the brain which thinks and visualizes the relation between oneself or oneself and others has been observed (Domhoff and Fox, 2015). Taken together, while these studies suggest that dreams can reflect aspects of the self, there is a severe lack of focal research from this perspective and further empirical research is necessary to unravel the validity of this connection.

This current research examines cultural differences in self-construal through agency in dream content between China, America and Japan. To elucidate the self-construal in dream content, we regard the dream-ego as the protagonist in the dream event while focusing on dream structures. These structures include the relationship between the dream-ego and other figures in the dream, and the extent of agency of the dream-ego, that is, its capacity to act or deal with challenges and the situation of the dream-ego by the end of the dream. To examine dream structures, we also regard dreams as narratives and use “Structural Dream Analysis (Roesler, 2018a, 2018b)” which adopts two methods for the analysis of narratives (Propp, 1975; Boothe, 1994).

Structural Dream Analysis categorizes dreams into the five general structural patterns found in pilot studies (Roesler, 2018b; Roesler et al., 2021). In addition, general structural patterns have the subclassifications. These subclassifications made up based on the dream ego’s initiative in relationships with others or capacity to deal with challenges in dream events, which capture the dreamer’s self-construal through agency in dream content. The detailed explanation of Structural Dream Analysis is presented in the method section below. Structural Dream Analysis originally aims to discuss common functions of dreams from the psychotherapeutic perspective and apply it to daily life for the non-clinical population. It focuses on the development and maturity of human psyche (Roesler, 2018a, 2018b) by identifying similarity between individuals’ dream series as the dreamers’ conditions improve through psychotherapy without other factors such as dreamers’ psychological symptoms. Therefore, it has not only been used for within-subject comparisons, also been shown to be suitable for between-group comparisons. In a meta-analysis on dreams of persons from Germany and Japan being in psychotherapy, Structural Dream Analysis revealed cultural differences in dreams between German and Japanese samples (Roesler et al., 2021). The cultural differences in the process of psychological recovery are thought to be caused by the difference between the East Asians’ self-formation process and the Western concept of “establishing the individual.” In addition, our previous study increased the sample size and compared dreams between the United States and Japan using Structural Dream Analysis, aiming to clarify the connection between dreams and cultural construal (Konakawa et al., 2023). The results indicated that American dreams tended to be more frequently classified into the subcategories where the dream-ego is active and takes initiative, compared to Japanese dreams. This suggests that both the degree of agency exhibited by the dream-ego and its relationship with other figures are implicitly influenced by culturally shaped models of the self—namely, the independent versus interdependent self-construal.

On the other hand, by comparing the dreams of Americans and Germans, there were also differences within the Westerners’ dreams. For Americans, dreams’ main theme was aiming for social success and status, but Germans’ dreams were less about such themes. Instead, their dreams often focused on taking action in difficult situations and achieving goals. Compared to Japan, the Americans and Germans are characterized by their strong agency and independence. However, even among Western countries, there may be cultural differences in the way the ego is oriented for social adaptation. There is a need to conduct comparisons even within East Asian countries, as this would allow us to examine a way of establishing identity in each culture based on dream structures.

### Anthropophobia mentality, sense of self and self-construal

In parallel with the dream structures, we also examine anthropophobia mentality, the sense of self and self-construal (independent and interdependent view of self), in an exploratory manner to compare participants’ cultural self-construal in this study.

Anthropophobia has been described as a typical neurosis among East Asians, particularly Japan, where it is closely related to *taijin kyofusho*—a cultural syndrome officially listed in the DSM-IV and DSM-5 under cultural concepts of distress (American Psychiatric Association, 1994, 2013). Anthropophobia is related to the fear of neighbors and acquaintances (people in intermediate relationships) or feeling threatened by the gaze of other persons (Nagata, 1992). Hence, the anthropophobia mentality scale (Horii, 2012) has been used in other research to assess sensitivity to others’ gaze and social evaluation, offering a culturally grounded index of interpersonal concerns that can inform cross-cultural studies of self-construal.

The concept of “sense of self” is also measured to capture the degree of integration of participants’ self (Matsuoka, 2015). By organizing and processing one’s psychological experience subjectively, the individual creates a unity called “self,” and the “sense of self” is defined as the subject responsible for this organizing action at a multilayered level such as through the senses, body, and language (Stern, 1985). Previous research has linked the sense of self to cultural patterns of independence and interdependence, using the scale to demonstrate how self-integration is experienced and valued differently across cultural contexts, with Western cultures typically valuing a strongly integrated sense of self.

East Asians with interdependent self-construal tend to focus on fitting in and being accommodating to others, because these acts give rise to pleasant, other-focused emotions (e.g., feeling of connection) while diminishing unpleasant ones (e.g., shame; Markus and Kitayama, 1991). This specifically taps into the degree that participants give weight on others’ perspective to define their self and has been conceptualized as the independent and interdependent view of self (Uchida, 2008). Views of self have been related to cultural models of independence and interdependence, providing a framework for interpreting how individuals prioritize autonomy versus connectedness in shaping their identity.

These concepts thus serve as a measure of the cultural differences in self-construal between West and East Asian cultures, forming the context to understand the interpretations of the dream structures in the current research.

### Overview of the current research

The present paper examines the cultural differences between Chinese, American and Japanese dreams, and the connection between independent and interdependent self-construal and dream structures. The current study replicates prior investigations conducted in Japan and the United States, extending the study to China, to highlight within culture differences in self-construal.

We predict that Chinese will show different patterns from both Americans and Japanese in independent and interdependent self-construal, as measured by the anthropophobia mentality scale (Horii, 2012), the sense of self scale (Matsuoka, 2015), and the independent and interdependent view of self scale (Uchida, 2008). Similar to previous studies, we posit that Americans will score higher in independent self-construal, and Chinese and Japanese will score higher in interdependent self-construal.

We also compare dreams across China, America, and Japan using Structural Dream Analysis, aiming to reveal the connection between dreams and cultural construal. Also, as previous studies on autobiographical memory have shown that childhood memories reflect cultural differences in self construal (Wang, 2001), childhood dreams can be examined from a similar perspective. Hence, this study examines “impressive dreams in childhood” as well as “recent impressive dreams.” This is not a longitudinal study of the development of dreams, but rather aims to examine the process of development of dreams using the dreamers’ subjective present view based on recalled dreams from childhood.

We hypothesize that Chinese dreams will more frequently depict initiative taken by other figures rather than the dream ego, compared to both American and Japanese dreams. This expectation stems from the assumption that, in Chinese culture, representations of others may be more salient and clearly defined—consistent with interdependent self-construal that emphasize relational clarity and social agency. In contrast, Japanese dreams may reflect a more diffuse or self-effacing relational style, where both self and others are less distinctly positioned. In that sense, this study offers further evidence that cultural milieu shapes the self both at the explicit and implicit level, because dreams represent one of the most extreme examples of “implicit” psychological processes.

## Materials and methods

### Study design

#### Participants

250 non-clinical participants from China were sampled through online platforms. During recruitment, participants were asked and filtered according to their age (above 18 years) and nationality. After removing incomplete responses, 173 participants (93 males, Mean age = 38.6 years, *SD* = 10.9) were left for analysis. Participants were informed that they were free to answer or decline the questionnaire, and that anonymity was assured. All data were analyzed anonymously. Demographic variables collected included participants’ age, gender, and (for the Chinese sample) residential region. Educational background was not collected for any of the samples. No notable biases were found in the distributions of age or gender across the three countries.

#### Procedure

The survey was conducted in May and June 2024 and was approved by the Ethical Review Committee for Clinical Psychology Research at Kyoto University. We commissioned Cross Marketing Inc. to recruit participants through online platforms. Participants signed up for a survey studying their daily experiences and dreams occurring during sleep. Participants’ demographics, target measures and dream content were then collected in the order as listed in the measures section below. Cases with missing data were excluded listwise for both the psychological scale data and the dream data prior to all analyses, including the Structural Dream Analysis. For cross-cultural comparisons, the Chinese data were analyzed alongside archival datasets from the United States and Japan reported by Konakawa et al. (2023). In those studies, participants were recruited through online platforms (Amazon Mechanical Turk in the United States and Lancers in Japan), and the surveys were conducted between June and August 2020. In this study, the term “childhood dreams” was operationally defined as dreams recalled from before age 18 (prior to high school graduation). Although this definition extends beyond the narrower age range often implied by “childhood,” it was adopted to ensure consistency with the survey instructions. The criterion was based on the fact that 18 is generally considered the age of adulthood in most regions of Japan, China, and the United States, and was applied consistently across all samples. Then, the data obtained from the Chinese sample were statistically compared with archival datasets from the United States (*n* = 220; 135 males; Mean age = 36.4, *SD* = 10.1) and Japan (*n* = 257; 146 males; Mean age = 39.9, *SD* = 10.6), as reported by Konakawa et al. (2023).

#### Measures

Participants completed the anthropophobia mentality scale (Horii, 2012), the sense of self scale (Matsuoka, 2015), the independent and interdependent view of self scale (Uchida, 2008) after answering items concerning dreams. The scales were translated from Japanese to Chinese by a professional translator. In addition, before coding, the free response for the dreams were also translated from Chinese to Japanese by the professional translator. A reliability test found an interrater agreement for the results coming from the same case of *k* = 0.79. and disagreements in coding were resolved based on discussion.

##### Dream content and structural dream analysis

Participants answered four items concerning dreams in a free response format to explain the contents of “impressive dreams in childhood,” “recent impressive dreams,” when these dreams occurred, and what happened in reality. For childhood dreams, participants were asked to recall their most memorable dream. Participants were asked to recall and describe an “impressive dream” from their childhood. This instruction was designed to elicit dreams that were personally meaningful or memorable, rather than to capture an exhaustive or objectively accurate record of childhood dreaming. This approach is consistent with narrative psychological methods that emphasize the subjective significance of recalled experiences. Participants were instructed to freely describe their dreams in an open-ended format, without any length requirement. This approach was chosen to preserve ecological validity and avoid imposing culturally biased constraints on narrative expression. Dream length (word count) was calculated using the Linguistic Inquiry and Word Count (LIWC) software for each language version separately (Chinese, Japanese, and English). Word counts were obtained based on the original language of the dream reports, prior to translation, to ensure that linguistic differences did not introduce artifacts in length comparison. This approach allows for a more valid cross-linguistic comparison of narrative length while preserving the natural linguistic structure of each sample. As mentioned above, this study examined participants recollections of dreams both from childhood and recently, where all participants wrote about both “impressive dreams during childhood” and “recent impressive dreams.” Given that the time the dreams occurred and what was concurrently happening in their real life were not the focal themes of the current study, participants’ free responses for these items are not included in the current analyses.

The coding for the free response for these dreams was done using Structural Dream Analysis. As mentioned above, Structural Dream Analysis categorizes dreams into the five general structural patterns found in pilot studies (Roesler, 2018b; Roesler et al., 2021). Structural Dream Analysis is a qualitative, interpretive research method that attempts to formalize the process of interpretation of the dream in a way that the conclusions are independent from the interpreter.

The five general structural patterns are shown in Table 1. The unit of analysis is the entire dream. One dream can be categorized by more than one pattern. For example, the first half of the dream could be classified as PatternI, but the latter half as Pattern II. Patterns II to V have subclassifications based on the relationship between the dream-ego and others in the dream, the extent of agency of the dream-ego, the end of the dream. One dream can contain two subclassification from the same variable pattern such as the subclassification of PatternII “The dream-ego is threatened by animals” and “The dream-ego is threatened by human beings” appeared in one dream (for more details, see Konakawa et al., 2023).

**Table 1:** The seven general structural patterns of structural dream analysis.

**PatternI** “No dream ego present” e.g., The dreamer just observes a scene as if watching a movie and does not actively take part in the dream. **PatternII** “The dream-ego is threatened” e.g., The dream-ego is threatened, attacked or injured and usually tries to escape or protect itself against the threatening figures. PatternIIa (subclassification): “The dream-ego is damaged, e.g., severely wounded, or even killed. In some cases the killing has already happened and the dream ego is found as a dead body” PatternIIb (subclassification): “The threat to the dream ego comes from a force in nature, e.g., a natural disaster, earthquake, fire, flooding, storm etc.” PatternIIc (subclassification): “The dream-ego is threatened by (dangerous) animals” PatternIId (subclassification): “The dream-ego is threatened by human beings, e.g., criminals, murderers or “evil people,” or human-like figures, e.g., ghosts, shadows etc.” PatternIII “The dream-ego is confronted with a performance requirement” e.g., The dream-ego is confronted with a performance requirement (examination, task to find something which was lost before, or produce something etc.), which is set by another figure or agency in the dream. PatternIIIa (subclassification): “Examination in a school or university setting” PatternIIIb (subclassification): “The dream ego is subject to an inspection by an official person, e.g., a ticket inspection on the train where the right of the dream ego is questioned” PatternIIIc (subclassification): “The dream-ego has the task to find something (which was lost before), get something, produce something etc.” **PatternIV** “Mobility dream: The dream-ego is moving towards a specified or unclear destination” e.g., The dream-ego is moving towards a specified or unclear destination, e.g., traveling and making use of different forms of transportation. PatternIVa (subclassification): “Disorientation: the dream ego has no idea where to go, even where it is and there are no signs of direction etc.” PatternIVb (subclassification): “The dream-ego is locked up in a closed space, imprisoned etc., and is looking for a way to get out” PatternIVc (subclassification): “The dream-ego wants to move, travel etc., but has no means to do so, e.g., it misses the train” PatternIVd (subclassification): “The dream-ego attempts to move and has some means of transportation but cannot control the movement, e.g., it cannot steer a car” PatternIVe (subclassification): “The dream-ego is moving but the way is blocked or the means of transport breaks down or crashes and movement cannot be continued” PatternIVf (subclassification): “The dream-ego is moving, making use of some means of transportation but it is going the wrong way, is in the wrong train or bus, or is not authorized to use it (e.g., has no ticket) and therefore cannot continue the journey” PatternIVg (subclassification):“In the positive form, the dream ego succeeds in moving towards and reaching the desired destination” **PatternV** “Social interaction dream: The dream-ego is occupied with making contact or communicating with others” e.g., The dream-ego wants to get in contact with another figure or attempts to communicate something to the other figure. PatternVa (subclassification):“The dream-ego wants to get into contact but is ignored by others” PatternVb (subclassification):“The dream-ego is criticized, devalued or made ridiculous by others and feels shame” PatternVc (subclassification):“The dream-ego is successful in creating the desired contact” PatternVd (subclassification):“A special case: the dream ego is aggressive towards others (even kills others) which expresses the will of the dream ego to be separated and autonomous” PatternVe (subclassification):“A special case: Positive behavior of others to the dream-ego”

The free responses for the dreams written in Chinese were translated into Japanese by a professional translator who is a native speaker of Japanese and bilingual in Chinese. Although a formal back-translation procedure was not conducted, translation accuracy was verified through professional translation and review by the research team. Dreams that were too short or too ambiguous to be reliably classified were excluded and coded as “unable to code”.

##### Anthropophobia mentality

The anthropophobia mentality scale (Horii, 2012) consists of 30 items measured using a 7-point Likert-scale, from 0 (Definitely not applicable) to 6 (Highly applicable). These items covered six components of anthropophobia mentality: I. Worries about oneself and others (e.g., “Become very anxious about how others think of me”), II. Worries about fitting into the group (e.g., “Cannot fit in well with a group”), III. Worries about social situations (e.g., “Feel intimidated in front of others”), IV. Worries about eye contact (e.g., “Cannot make eye contact with others”), V. Worries about controlling oneself (e.g., “Give up easily due to lack of patience”), and VI. Worries about living (e.g., “Cannot find meaning in life”). The anthropophobia mentality scale strongly reflects participants’ weight of emphasis and concerns about social interaction, which has been a strong contributor to the differences between independent and interdependent self-construal (Kitayama et al., 2006; Uchida et al., 2009). The internal consistency of the scale was excellent across all three countries, with Cronbach’s *α* coefficients of 0.97 for Japan, 0.98 for the United States, and 0.98 for China. These values indicate highly reliable measurement across cultural contexts (see Table 2 for details).

**Table 2:** Cronbach’s *α* coefficients for each scale by country.

Scale	Chinese	American	Japanese
Anthropophobia mentality	0.98	0.98	0.97
Sense of self	0.84	0.89	0.85
Independent view of self	0.84	0.81	0.73
Interdependent view of self	0.80	0.71	0.70

##### Sense of self

The sense of self scale (Matsuoka, 2015) consists of 23 items measured using a 5-point Likert-scale, from 1 (not at all) to 5 (frequently). These items covered four components of the sense of self: sense of core self (e.g., “Unsure of the range of my body”), sense of emotion self (e.g., “Cannot verbalize my feelings well”), sense of mindreading self (e.g., “Am aware of hidden intentions in actions of others”; reverse scored item), and sense of verbal/narrative selves (e.g., “Unconsciously tell a lie or a made-up story during conversation”). The internal consistency of the scale was good across all three countries, with Cronbach’s *α* coefficients of 0.85 for Japan, 0.89 for the United States, and 0.84 for China. These values suggest that the scale demonstrated stable reliability across cultural groups (see Table 2 for details).

##### Independent and interdependent view of self

The independent and interdependent view of self scale (Uchida, 2008) consists of 20 items measured using a 5-point Likert-scale, from 1 (does not describe me at all) to 5 (describes me very much). These items covered two components of the scale: independent view of self (10 items; e.g., “I always try to have my own opinions”) and interdependent view of self (10 items; e.g., “I am concerned about what people think of me”). The internal consistency of the independent and view of self scale was acceptable in the Japanese sample (α = 0.73), and good in the American (α = 0.81) and Chinese (α = 0.84) samples (see Table 2 for details). The internal consistency of the interdependent view of self scale was acceptable in the Japanese (α = 0.70) and American (α = 0.71) samples, and good in the Chinese sample (α = 0.80) (see Table 2 for details). This instrument was selected to ensure consistency with prior data from Japan and the United States, allowing for direct cross-cultural comparison. While the Singelis (1994) scale is more widely used internationally, the Uchida scale has been validated in East Asian contexts and was deemed appropriate for the present study.

### Statistical analysis

#### Mixed-effects logistic regression

We used logistic mixed-effects regression to examine the effects of time (childhood, recent), country (US, Japan, China), word count (WC), age, and gender on the likelihood of reporting each dream category. The general model structure was:

$\text{logit}(P(Y_{\mathrm{ij}}=1))=\beta_{0}+\beta_{1}{\text{Time}}_{\mathrm{ij}}+\beta_{2}{\text{Country}}_{i}+\beta_{3}{\mathrm{WC}}_{i}+\beta_{4}{\mathrm{Age}}_{i}+\beta_{5}{\text{Gender}}_{i}+u_{i}$ where *Y*_ij_ is the binary indicator of whether participant *i* reported category *j*, and u_i_ is a random intercept accounting for repeated measures across time within participants. Time was treated as a within-subject factor, while country, WC, age, and gender were between-subject predictors.

Odds ratios (OR) and FDR-adjusted *p*-values are reported in the main text; full regression coefficients (*β*), standard errors, and z-values are provided in Supplementary Table S1.

## Results

### Cross-cultural comparisons in dream descriptives

#### Dream length

A one-way ANOVA revealed a significant effect of country on length of dream record for “impressive dreams in childhood,” *F* (2, 647) = 119.19, *p* < 0.001, *η^2^* = 0.27. *Post hoc* comparisons using Tukey’s HSD test indicated that Chinese participants’ dreams (*M* = 10.28, *SD* = 13.46) were significantly shorter than American participants’ dreams (*M* = 53.02, *SD* = 37.82), *p* < 0.001; and Chinese participants’ dreams (*M* = 10.28, *SD* = 13.46) were significantly shorter than Japanese participants’ dreams (*M* = 21.09, *SD* = 28.52), *p* < 0.001. In addition, a one-way ANOVA revealed a significant effect of country on length of dream record for “recent impressive dreams,” *F* (2, 647) = 76.02, *p* < 0.001, *η^2^* = 0.19. Post hoc comparisons using Tukey’s HSD test indicated that Chinese participants’ dreams (*M* = 9.28, *SD* = 14.56) were significantly shorter than American participants’ dreams (*M* = 52.36, *SD* = 49.79), *p* < 0.001; and Chinese participants’ dreams (*M* = 9.28, *SD* = 14.56) were significantly shorter than Japanese participants’ dreams (*M* = 20.65, *SD* = 33.83), *p* = 0.005.

#### When “impressive dreams in childhood” occurred

Many participants could not recall the exact age the dream occurred and described it “in 〇〇 school days.” Other participants reported their recurring dreams in expressions such as “happened many times in elementary school days.” In addition, participants who reported dreams occurring after senior high school days were removed from the analyses. Therefore, the data was organized in the following form. For Chinese participants, 29 dreams occurred during preschool days, 110 dreams occurred during elementary school days, 23 dreams occurred during junior high school days, 11 dreams occurred during senior high school days. A chi-squared test was used to determine the differences in distribution when “impressive dream in childhood” occurred across countries. Comparisons among Chinese, American and Japanese participants when “impressive dreams in childhood” occurred did not reach significance, *χ^2^* (6) = 7.07, *p* = 0.314, Cramer’s V = 0.074.

#### When “recent impressive dreams” occurred

Many participants could not recall the exact date the dream occurred and described it as “〇 weeks/months/year ago” or “last week/month/year.” Other participants reported their recurring dreams in expressions such as “happened many times in the past 〇 months/years.” In addition, participants who did not report when their dreams occurred were removed from the analyses. Therefore, the data was organized in the following form. For Chinese participants, 87 dreams occurred within a week, 43 dreams occurred within a month, 34 dreams occurred within a year, 9 dreams occurred within five years. A chi-squared test was used to determine the differences in distribution when “recent impressive dream” occurred across countries. Comparisons among Chinese, American and Japanese participants when “recent impressive dream” occurred reached significance, *χ^2^* (6) = 21.79, *p* = 0.001, Cramer’s V = 0.129. Residual analysis revealed that Chinese participants had significantly more reported dreams occurring within a week (adjusted residual = 4.0), while there were significantly less reported dreams occurring within a year (adjusted residual = −2.4). Whereas, American participants had significantly less reported dreams occurring within a week (adjusted residual = −2.0), while there were significantly more reported dreams occurring within a month (adjusted residual = 2.5). Additionally, the Japanese sample was significantly more reported dreams occurred within a year (adjusted residual = 2.5). These findings suggest that Chinese participants exhibit a different pattern of categorical distribution compared to the other groups, particularly for dreams occurring within a week and within a year.

### Cross-cultural comparisons in dream structure

To evaluate cross-cultural differences in dream structure, we implemented mixed-effects logistic regression models with random intercepts for participants, including country as the main predictor and covariates for time, WC, age, and gender. False discovery rate (FDR) correction was applied across the family of subcategory tests to control for multiplicity. This model allows us to rigorously identify cultural differences while controlling for potential confounding factors such as narrative length (WC), dream type (time), age, and gender.

Statistically significant results from the mixed-effects logistic regression (China as the reference category) are presented in Table 3.

**Table 3:** Mixed-effects logistic regression predicting dream features (China as reference category, significant results only).

Feature	Predictor	Estimate (SE)	z	FDR *p*
PatternIIa	Country US	1.33 (0.44)	3.00	0.0098**
	Country Japan	2.41 (0.00)	517.68	<0.001***
PatternIId	Country US	1.10 (0.31)	3.55	0.0019**
	Country Japan	0.85 (0.30)	2.83	0.016*
PatternII	Country US	0.87 (0.24)	3.64	0.0014**
	Country Japan	0.75 (0.22)	3.34	0.0034**
PatternIIIa	Country Japan	−2.22 (0.84)	−2.63	0.028*
PatternIIIc	Country US	−1.36 (0.37)	−3.65	0.0013**
PatternIII	Country US	−1.73 (0.34)	−5.15	<0.001***
	Country Japan	−0.99 (0.29)	−3.42	0.0027**
PatternIVa	Time Recent	−1.39 (0.44)	−3.14	0.0064**
PatternIV	Time Recent	−0.86 (0.25)	−3.44	0.0026**
PatternVc	Country Japan	−0.85 (0.35)	−2.41	0.049*
PatternVe	Country US	−1.55 (0.37)	−4.18	<0.001***
PatternV	Time Recent	0.78 (0.24)	3.20	0.0053**

Estimates are log-odds coefficients. SE = standard error. *p* values are FDR-adjusted. The significance *p* < 0.001 was marked by ***, *p* < 0.01 was marked by **, *p* < 0.05 by *.

Country effects were the most consistent, underscoring robust cross-cultural differences in dream structure. Specifically, American and Japanese participants were more likely than Chinese participants to report following features; for PatternIIa, American participants (OR = 3.78, FDR-adjusted *p* = 0.0098) and Japanese participants (OR = 11.12, FDR-adjusted *p* < 0.001) were more likely than Chinese participants to report “the dream-ego is damaged.” For PatternIId, American participants (OR = 3.00, FDR-adjusted *p* = 0.0019) and Japanese participants (OR = 2.34, FDR-adjusted *p* = 0.016) were more likely than Chinese participants to report “the dream-ego is threatened by human beings.” For PatternII, American participants (OR = 2.39, FDR-adjusted *p* = 0.0014) and Japanese participants (OR = 2.12, FDR-adjusted *p* = 0.0034) were more likely than Chinese participants to report “the dream-ego is threatened.” Conversely, American and Japanese participants were less likely than Chinese participants to report following features; For PatternIIIa, Japanese participants (OR = 0.11, FDR-adjusted *p* = 0.028) were less likely than Chinese participants to report “examination.” For PatternIIIc, American participants (OR = 0.26, FDR-adjusted *p* = 0.0013) were less likely than Chinese participants to report “the dream-ego has the task to find something (which was lost before), get something, produce something etc.”. For PatternIII, American participants (OR = 0.18, FDR-adjusted *p* < 0.001) and Japanese participants (OR = 0.37, FDR-adjusted *p* = 0.0027) were less likely than Chinese participants to report “the dream-ego is confronted with a performance requirement.” For PatternVc, Japanese participants (OR = 0.43, FDR-adjusted *p* = 0.049) were less likely than Chinese participants to report “the dream-ego is successful in creating the desired contact.” For PatternVe, American participants (OR = 0.21, FDR-adjusted *p* < 0.001) were less likely than Chinese participants to report “positive behavior of others to the dream-ego”.

Beyond the effect of country, temporal and individual covariates also showed effects. Dream Type (time) was associated with reduced incidence in PatternsIVa and IV, but increased incidence in PatternV. Narrative length (WC) exhibited small positive associations in several categories, and age showed minor negative effects. Gender effects were generally weak or unstable. Taken together, these findings highlight country as the most robust predictor of structural dream features, supporting the validity of conducting international comparisons of dream structure.

In Chinese dreams, the dream-ego was typically placed in situations such as exams (examples 1–1 and 1–2) or getting something (examples 1–3 and 1–4), and showed a marked tendency to engage passively with spontaneously arising tasks. Situations in which the dream-ego was threatened appeared less frequently than in American or Japanese dreams. In American dreams, by contrast, the dream-ego exhibited strong agency and was depicted confronting threats (examples 2–1 and 2–2). In Japanese dreams, the dream-ego tended to fade passively from the dream, indicating the fragility of agency (examples 3–1 and 3–2) (see Table 4).

**Table 4:** Examples of the structural patterns identified in Chinese, American and Japanese participants for “Impressive dreams in childhood” and “Recent impressive dreams”.

Example 1-1, 2 of the situation that “Examination in a school or university setting” (of III): in Chinese participants “*I got the top score in the test.*” (impressive dreams in childhood) “I was back to being a student, *taking the Gaokao in an exam hall.”* (recent impressive dreams)
Example 1-3, 4 of the situation that “The dream-ego has the task to find something (which was lost before), get something, produce something etc.” (of III): in Chinese participants “*I caught fish in the mountain.*” (impressive dreams in childhood) “The most impressive dream I saw recently was that *I caught a huge black carp weighing 68 jin*.” (recent impressive dreams)
Example 1-5, 6 of the situation that “The dream-ego is successful in creating the desired contact” (of V): in Chinese participants “Because I got a good score on the Gaokao for university admissions, *my whole family was proud of me.*” (impressive dreams in childhood) “*I got a job at the company I had hoped to work for*.” (recent impressive dreams)
Example 1-7, 8 of the situation that “Others take the initiative in positive behavior to the dream-ego” (of V): in Chinese participants “It was New Year’s. *I was preparing for the New Year with my family, eating dumplings, and playing with firecrackers.*” (impressive dreams in childhood) “*A lot of relatives visited my house.*” (recent impressive dreams)
Example 2-1, 2 of the situation that “The dream-ego is threatened by human beings” (of II): in American participants “I was in church. *There was a group of people there with guns.* We had guns as well. We hid behind a bench and shot back at them.” (impressive dreams in childhood) “I was walking around and found lots of crystals coming up from the ground. *There were these two strangers that were eyeing me from a distance, later on they approached me and snatched my bag.*” (recent impressive dreams)
Example 2-3, 4 of the situation that “The dream-ego is successful in creating the desired contact” (of V): in American participants “I remember being in the sky and being able to fly. *I did my best trying to control my flight and seeing the world and impressing my friends.*” (impressive dreams in childhood) “The most memorable dream I had recently was when I was competing a basketball game. The game was tied, *I took the last shot and made it in. Everyone on my team was happy and celebrated with me winning the game.*” (recent impressive dreams)
Example 3-1, 2 of the situation that “The dream-ego is damaged, e.g., severely wounded, or even killed.” (of II): in Japanese participants “*I had a dream in which I was run over and killed on a Shinkansen track*. My disembodied self was floating above and looking down at my own body.” (impressive dreams in childhood) “*A person whose face I could not see stabbed me in the abdomen with a blade*. Blood gushed out.” (recent impressive dreams)
Example 3-3, 4 of the situation that “Others take the initiative in positive behavior to the dream-ego” (of V): in Japanese participants “In the corner of a tatami room, *a bob-haired girl was looking at me and beckoning to me.*” (impressive dreams in childhood) “*I was going home with a classmate from high school*.” (recent impressive dreams)

In addition, the distribution of subclassifications within the structural pattern “The dream ego is occupied with making contact or communicating with others” (V) varied markedly across Chinese, American, and Japanese dreams. In American dreams, the dream-ego exhibited strong agency, acting deliberately in relationships with others and experiencing social achievement (examples 2–3 and 2–4). By contrast, Japanese dreams frequently depicted the dream-ego and others as simply being together without clear direction (examples 3–3 and 3–4), with the dream-ego often sharing agency with others. In Chinese dreams, both themes of social recognition (examples 1–5 and 1–6) and coexistence with friendly others, particularly family members (examples 1–7 and 1–8), tended to appear (see Table 4).

While Japanese dreams tended to depict passivity in a more diffused or self-effacing way, Chinese dreams suggested that, although the dream-ego may lack a strong confrontational self, it engages passively with social relationships in a positive and affirming manner rather than simply experiencing external pressure or constraint.

### Cross-cultural comparisons in self-construal

Multi-group confirmatory factor analyses (CFA) were conducted to examine measurement invariance of the four scales (anthropophobia, sense of self, independent self, and interdependent self) across the three cultural groups. Configural invariance was poor to marginal for most scales, and subsequent metric and scalar models showed further deterioration in fit indices (see Supplementary Table S2). As a result, direct cross-group mean comparisons were not fully supported.

Instead, we focused on within-group correlations and patterns of associations to interpret cultural differences. By examining these associations, distinct cross-cultural patterns emerge, suggesting that the constructs may be embedded differently within the psychological systems of each cultural group. To illustrate these patterns, Tables 5–7 present the correlations among the measures within each sample.

**Table 5:** The correlation among each of these measures to one another in the American participants.

Measure	1	2	3	4
1. Anthropophobia mentality	–			
2. Sense of self	−0.59***	–		
3. Independent view of self	−0.56***	0.43***	–	
4. Interdependent view of self	0.38***	−0.18**	−0.32***	–

The significance *p* < 0.001 was marked by ***, *p* < 0.01 was marked by **.

**Table 6:** The correlation among each of these measures to one another in the Japanese participants.

Measure	1	2	3	4
1. Anthropophobia mentality	-			
2. Sense of self	−0.50***	-		
3. Independent view of self	−0.38***	0.28***	-	
4. Interdependent view of self	0.15*	−0.00	−0.29***	-

The significance *p* < 0.001 was marked by ***, *p* < 0.05 by *.

**Table 7:** The correlation among each of these measures to one another in the Chinese participants.

Measure	1	2	3	4
1. Anthropophobia mentality	-			
2. Sense of self	−0.50***	–		
3. Independent view of self	−0.14	0.28***	-	
4. Interdependent view of self	0.15	−0.04	0.39***	-

The significance *p* < 0.001 was marked by ***.

In the Chinese sample, independent and interdependent self-construals were positively correlated (*r* = 0.39, *p* < 0.001), suggesting that these dimensions may coexist rather than oppose each other. In contrast, they were negatively correlated in the American (*r* = −0.32, *p* < 0.001) and Japanese samples (*r* = −0.29, *p* < 0.001). Furthermore, in the American and Japanese samples, higher independent self-construal was associated with lower anthropophobia (American: *r* = −0.56, *p* < 0.001; Japanese: *r* = −0.38, *p* < 0.001), whereas higher interdependent self-construal was associated with higher anthropophobia (American: *r* = 0.38, *p* < 0.001; Japanese: *r* = 0.15, *p* = 0.014). No such associations were observed in the Chinese sample (independent: *r* = −0.14, *p* = 0.064; interdependent: *r* = 0.15, *p* = 0.056), indicating that self-construal may not directly predict anthropophobia in this cultural context.

These findings highlight culturally distinct models of the self and suggest that the influence of self-construal on emotional experience may vary across societies, with implications for understanding the interplay between individual and collective aspects of self in different cultural settings.

## Discussion

The current research examined cultural differences among Chinese, American, and Japanese dreams, while also exploring how these dream structures may be associated with broader cultural self-construal. To contextualize these findings, we first measured participants’ independent and interdependent views of self (Uchida, 2008), anthropophobia mentality (Horii, 2012), and sense of self (Matsuoka, 2015), which together provided indices of how individuals define and integrate the “self” within their cultural context.

As shown in the correlational analyses, culturally distinct patterns emerged in the relationships between self-construal and anthropophobia. In the Chinese sample, independent and interdependent self-construals were positively correlated, suggesting that these orientations may coexist rather than oppose each other. In contrast, they were negatively correlated in the Japanese and American samples, consistent with the view that independence and interdependence represent opposing orientations in these cultural contexts.

Moreover, in Japan and the United States, higher independent self-construal was associated with lower anthropophobia, whereas higher interdependent self-construal was associated with higher anthropophobia. No significant associations were observed in the Chinese sample, indicating that self-construal may not directly predict anthropophobia in this cultural context. Taken together, these findings suggest that the psychological meaning of independence and interdependence differs across societies: in China, the two orientations may be integrated within the same self-system, while in Japan and the United States they appear to function in a more antagonistic manner.

Within this framework, we then examined dream content as an implicit expression of self-construal. Differences in dream structure across the three cultures were broadly consistent with the scale findings, suggesting association between self-related processes and their expression in unconscious, narrative form.

The primary contribution of this study lies in illustrating potential associations between dream structures and self-construal across cultures, including nuanced variations within East Asia. By comparing Chinese, Japanese, and American participants, the study highlights both broad cultural contrasts and more subtle divergences between neighboring East Asian societies.

Consistent with prior research on self-construal, American participants’ dreams tended to emphasize individual agency and achievement, often featuring themes of proactive behavior, personal success, or the dream ego overcoming obstacles. In contrast, dreams from Japan and China reflected a more interdependent and relational orientation, with the dream ego often appearing in connection with others. These patterns are consistent with longstanding findings that East Asian cultural contexts are characterized by relational self-construal, whereas American cultural contexts tend to emphasize an independent orientation (Markus and Kitayama, 1991).

However, our findings also indicated an important distinction between Japan and China. Although both cultures displayed a more passive and relational stance in their dreams, the tone and meaning of this passivity differed. In Japanese dreams, passivity frequently appeared in diffused or self-effacing forms-such as the dream-ego being harmed or even disappearing into others-suggesting that merging with the group may sometimes come at the cost of personal agency or clarity of self. By contrast, Chinese dreams, while also often relational and passive, typically portrayed the presence of others in a positive, affirming light. Rather than expressing strong confrontational agency, the dream-ego was supported and validated by friendly figures, implying that Chinese participants may maintain their foundation of selfhood by moving toward and integrating with affirming others, thereby cultivating a shared sense of humanity rather than merely merging into the group.

In terms of Chinese dreams, themes of “examinations” and “working hard to complete assigned tasks” were especially prominent, even in adulthood. This pattern may reflect China’s dual orientation: a relatively high independent self-construal, similar to Americans, alongside an interdependent self-construal shared with East Asian cultures. Consistent with Zhao et al. (2024), who reported that familism scores—reflecting bonds and support within family networks—are slightly higher in China compared to Japan and the United States, our findings suggest that the Chinese self may develop not only through relationships but also through striving toward an ideal, often framed by social expectations. These dreams often conveyed a sense of pressure, yet in a context where others’ presence felt supportive rather than merely constraining.

These findings are in line with discussions on cultural differences in conceptions of happiness and interpersonal relationships (Uchida et al., 2004; Uchida and Ogihara, 2012). They indicate that even within a broadly interdependent framework, there are distinct psychological pathways: Japanese dreams portray a relational self that sometimes dissolves into the collective, while Chinese dreams portray a relational self-sustained by positive social ties. Together, these results underscore that “East Asia” is not monolithic—shared interdependence manifests differently across cultural contexts, with important implications for understanding how self-construal is formed and expressed in both waking life and dreams.

This study has several limitations that should be considered when interpreting the results. First, the use of previously collected data for some cultural groups may introduce temporal inconsistencies, as cultural dynamics can shift over time. Although efforts were made to ensure comparability across datasets by applying consistent data processing methods and careful cultural interpretation, longitudinal consistency cannot be fully guaranteed; therefore, the results should be interpreted with appropriate caution.

Second, while professional translation services were employed for the Chinese scales and expert review was conducted, back-translation procedures were not performed to fully verify equivalence. This may affect the validity of the self-report data in the Chinese sample. Future research will incorporate back-translation and pilot testing to enhance the validity and reliability of translated instruments.

Third, coders were not fully blind to the country of origin of each dream, although the Chinese dream reports were translated into Japanese before coding to minimize language-related bias. Moreover, there were no formal manualized rules for handling very short or highly ambiguous dreams. Such cases were excluded and coded as “unable to code.” Future studies should establish clearer coding criteria and conduct cross-language calibration to further improve coding reliability.

In addition, as noted earlier, a further implication of the measurement invariance analyses is that some of the self-report scales may not fully capture equivalent constructs across cultures. For example, the anthropophobia scale may tap into somewhat different images or experiences in China compared to Japan or the United States. Rather than treating this solely as a limitation, our findings highlight the need for developing culturally appropriate instruments that can more validly support cross-national comparisons. Demonstrating this need represents an important contribution of the present study, as it points to a concrete direction for future research in cross-cultural dream analysis and personality assessment.

Finally, we note that differences in dream length and self-disclosure norms across online platforms may have influenced participants’ descriptions of dreams. These factors should be considered as possible alternative explanations for the observed cultural differences. Although the study examined three distinct cultural groups, East Asia itself is culturally diverse, and further research is needed to explore intra-regional differences beyond China and Japan. Future research is planned on expanding international comparisons and surveying long-term residents and immigrants. By examining how people adjust to different cultural contexts, and what remains stable, we can get a better understanding of psychological well-being and how to deal with psychological adjustment and well-being in East Asia.

## Data Availability

The datasets presented in this article are not readily available because the datasets generated and analyzed during the current study are not publicly available due to privacy and ethical restrictions, as they contain sensitive information from participants’ responses to questionnaires and dream reports. Requests to access the datasets should be directed to HK, konakawa.hisae.8e@kyoto-u.ac.jp.
