# Supplementary material for: Examining the association between cultural self-construal and dream structures in China, Japan, and the United States
Source: Front Psychol. 2026 Jan 12;16:1688407. doi: 10.3389/fpsyg.2025.1688407 (PMC12832506; doi:10.3389/fpsyg.2025.1688407)
Supplement: Supplementary file 1 [file Data_Sheet_1.docx]

**Supplementary Material 4: Representative Dream Excerpts**

This document provides representative dream excerpts from Chinese, American, and Japanese participants, selected to illustrate culturally distinct patterns of interpersonal contact (Pattern V). Each excerpt is anonymized and translated into English with minimal interpretation to preserve original narrative tone.

**1. Chinese dreams**

Example 1. 【childhood dream】

“I was preparing for the New Year with my family, enjoying dumplings and playing with firecrackers.”

Example 2. 【childhood dream】

“I went to the zoo with my whole family.”

Example 3. 【recent dream】

“I met my older child, and they made a bracelet for me.”

Example 4. 【recent dream】

“I ate dumplings together with my whole family, and we had a great time.”

**2. American dreams**

Example 1. 【childhood dream】

“I scoring the winning goal in the world cup. All of my teammates pig piled on me. It was the greatest moment of my life.”

Example 2. 【childhood dream】

“I was an airline pilot. I traveled around the world and was admired by many.”

Example 3. 【recent dream】

“I ran for president and won the election.”

Example 4. 【recent dream】

“The most memorable dream I had recently was when I was competing a basketball game. The game was tied, I took the last shot and made it in.”

**3. Japanese dreams**

Example 1. 【childhood dream】

“I was riding a train with my late grandmother. I asked, ‘Why are you here?’ and she said, ‘I felt a little lonely, so I came.’ I thought, ‘I see,’ and kept swaying with the train.”

Example 2. 【childhood dream】

“I was on a bus, and a classmate was sitting next to me. We were friendly, but not close enough to be riding a bus together, so I wondered why they appeared in my dream.”

Example 3. 【recent dream】

“I was going home with a classmate from high school.”

Example 4. 【recent dream】

“I was practicing with my college friends in a rented studio, playing in a rock band. They were dressed just like they used to be, playing with full concentration, and I was strumming my guitar along with them, feeling really good. I remember being filled with a nostalgic feeling.”
