# Supplementary material for: Examining the association between cultural self-construal and dream structures in China, Japan, and the United States
Source: Front Psychol. 2026 Jan 12;16:1688407. doi: 10.3389/fpsyg.2025.1688407 (PMC12832506; doi:10.3389/fpsyg.2025.1688407)
Supplement: Supplementary file 2 [file Data_Sheet_2.docx]

**Supplementary Material 5: Item Characteristics and Exploratory Factor Analysis**

**Item characteristics**

Means, standard deviations, corrected item–total correlations, and Cronbach’s alpha-if-item-deleted statistics for all 23 items are presented in Table S5. Corrected item–total correlations ranged from .163 to .634. Cronbach’s alpha for the full scale was .877, and no item deletion improved reliability, indicating that all items contributed adequately to internal consistency.

**Exploratory factor analysis**

An exploratory factor analysis (EFA) was conducted to examine the latent structure of the 23-item Sense of Self scale. The Kaiser–Meyer–Olkin (KMO) measure indicated excellent sampling adequacy (KMO = .916), and Bartlett’s test of sphericity was significant, χ² (253) = 5884.70, p < .001, confirming that the correlation matrix was suitable for factor analysis.

Principal axis factoring with oblimin rotation extracted four factors with eigenvalues greater than 1, accounting for 55.5% of the total variance prior to rotation. The scree plot (Fig. S1) showed a marked break after the first one to two factors, suggesting that a more parsimonious structure (e.g., two factors) may also be plausible.

Factor 1 consisted of items reflecting a coherent, stable sense of self (e.g., q15r, q20r, q22r, q21r), whereas Factor 2 comprised items representing conflicted or negatively valenced self-perceptions (e.g., q13r, q11r, q05r). The remaining factors showed mixed or cross-loading items, making them difficult to interpret conceptually (Table S6).

Communalities ranged from .26 to .72, with several items (e.g., q06r, q09r, q18r) exhibiting relatively low commonality (< .30). Because internal consistency did not improve with item removal, all items were retained for the analyses reported in the main text.

**Supplementary Table S5.** Item descriptive statistics

| **Item** | **Mean** | **SD** | **Corrected item–total correlation** | **α if item deleted** |
| --- | --- | --- | --- | --- |
| q01r | 3.45 | .889 | .316 | .875 |
| q02r | 3.92 | .851 | .619 | .867 |
| q03r | 3.99 | 1.054 | .589 | .867 |
| q04r | 3.66 | 1.191 | .577 | .867 |
| q05r | 3.56 | .936 | .308 | .875 |
| q06r | 3.27 | 1.122 | .329 | .875 |
| q07r | 3.93 | 1.036 | .592 | .867 |
| q08r | 3.97 | 1.055 | .514 | .869 |
| q09r | 4.39 | .983 | .447 | .871 |
| q10r | 3.76 | 1.034 | .395 | .873 |
| q11r | 3.61 | 1.009 | .293 | .876 |
| q12r | 3.92 | .996 | .575 | .868 |
| q13r | 3.19 | 1.027 | .240 | .877 |
| q14r | 3.85 | 1.045 | .634 | .866 |
| q15r | 4.24 | 1.012 | .569 | .868 |
| q16r | 3.15 | 1.048 | .163 | .880 |
| q17r | 3.77 | 1.099 | .590 | .867 |
| q18r | 3.90 | 1.039 | .468 | .871 |
| q19r | 3.44 | 1.022 | .423 | .872 |
| q20r | 4.41 | .948 | .524 | .869 |
| q21r | 4.08 | .965 | .529 | .869 |
| q22r | 4.41 | .925 | .521 | .869 |
| q23r | 3.30 | 1.049 | .260 | .877 |

**Supplementary Table S6.** Factor loadings

| **Item** | **Factor 1** | **Factor 2** | **Factor 3** | **Factor 4** |
| --- | --- | --- | --- | --- |
| q22r | .756 | – | – | – |
| q15r | .742 | – | – | – |
| q20r | .728 | – | – | – |
| q21r | .636 | – | – | – |
| q08r | .495 | – | – | – |
| q10r | .430 | – | – | – |
| q12r | .425 | – | – | – |
| q09r | .412 | – | – | – |
| q02r | .404 | – | – | – |
| q13r | – | .733 | – | – |
| q11r | – | .719 | – | – |
| q05r | – | .699 | – | – |
| q19r | – | .652 | – | – |
| q23r | – | .628 | – | – |
| q16r | – | .602 | – | – |
| q01r | – | .554 | – | – |
| q04r | – | – | –.929 | – |
| q17r | – | – | –.741 | – |
| q03r | – | – | –.721 | – |
| q14r | – | – | –.551 | – |
| q06r | – | – | .329 | – |
| q07r | – | – | –.307 | .326 |
| q18r | – | – | – | – |

**Figure S1.** Scree plot


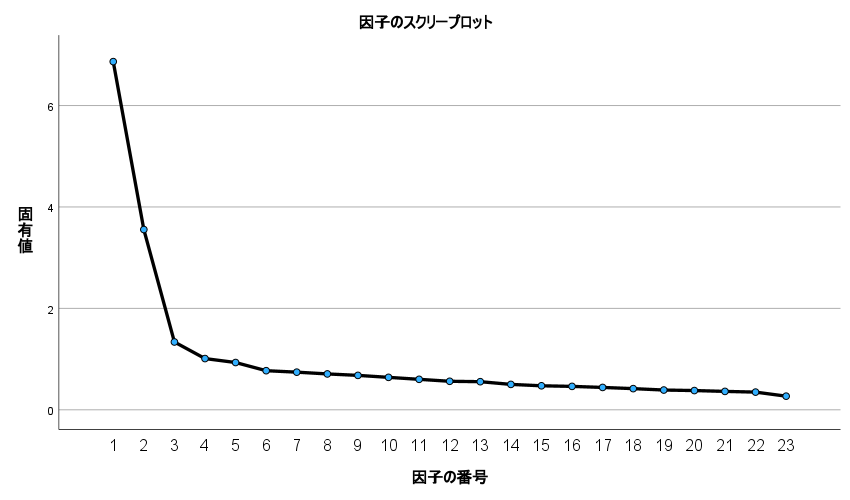


Factor Number

Scree Plot of Factors

Eigenvalue
