# Supplementary material for: Examining the association between cultural self-construal and dream structures in China, Japan, and the United States
Source: Front Psychol. 2026 Jan 12;16:1688407. doi: 10.3389/fpsyg.2025.1688407 (PMC12832506; doi:10.3389/fpsyg.2025.1688407)
Supplement: Supplementary file 3 [file Table_1.docx]

**Supplementary Material 1: the mixed-effects logistic regression (China as reference category)**

**Supplementary Table S1**

| **Feature** | **Term** | **regression coefficients (β)** | **Std. Error** | **z value** | **Pr(>\|z\|)** | **FDR_P** |
| --- | --- | --- | --- | --- | --- | --- |
| PatternⅠ | (Intercept) | -3.04 | 0.61 | -4.98 | <0.001 | <0.001 |
| PatternⅠ | Country US | -1.83 | 0.85 | -2.17 | 0.030 | 0.082 |
| PatternⅠ | Country Japan | 0.95 | 0.40 | 2.36 | 0.018 | 0.055 |
| PatternⅠ | Dream Type (time) | -0.44 | 0.54 | -0.81 | 0.417 | 0.653 |
| PatternⅠ | Narrative Length (WC) | -0.01 | 0.009 | -1.36 | 0.173 | 0.367 |
| PatternⅠ | Age | 0.004 | 0.01 | 0.31 | 0.755 | 0.921 |
| PatternⅠ | Gender | 0.22 | 0.31 | 0.72 | 0.473 | 0.719 |
| PatternⅠ | Gender Female | 0.54 | 0.66 | 0.82 | 0.413 | 0.652 |
| PatternⅠ | Gender Male | -0.29 | 0.69 | -0.42 | 0.673 | 0.872 |
| PatternⅡa | (Intercept) | -4.96 | 0.005 | -1058.28 | 0 | 0 |
| PatternⅡa | Country US | 1.33 | 0.44 | 3.00 | 0.002 | 0.010 |
| PatternⅡa | Country Japan | 2.41 | 0.005 | 517.68 | 0 | 0 |
| PatternⅡa | Dream Type (time) | -0.40 | 0.005 | -85.13 | 0 | 0 |
| PatternⅡa | Narrative Length (WC) | 0.001 | 0.003 | 0.36 | 0.720 | 0.893 |
| PatternⅡa | Age | -0.07 | 0.004 | -18.86 | <0.001 | <0.001 |
| PatternⅡa | Gender | -0.82 | 0.005 | -177.75 | 0 | 0 |
| PatternⅡa | Gender Female | -0.11 | 0.51 | -0.22 | 0.828 | 0.967 |
| PatternⅡa | Gender Male | -0.45 | 0.005 | -96.00 | 0 | 0 |
| PatternⅡb | (Intercept) | -7.04 | 1.81 | -3.89 | <0.001 | <0.001 |
| PatternⅡb | Country US | 0.43 | 0.92 | 0.47 | 0.641 | 0.849 |
| PatternⅡb | Country Japan | 1.13 | 0.90 | 1.26 | 0.208 | 0.413 |
| PatternⅡb | Dream Type (time) | -0.89 | 1.11 | -0.80 | 0.425 | 0.661 |
| PatternⅡb | Narrative Length (WC) | 0.01 | 0.006 | 2.28 | 0.023 | 0.067 |
| PatternⅡb | Age | -0.01 | 0.04 | -0.27 | 0.790 | 0.948 |
| PatternⅡb | Gender | -0.28 | 0.71 | -0.39 | 0.697 | 0.891 |
| PatternⅡb | Gender Female | -0.69 | 1.44 | -0.48 | 0.634 | 0.845 |
| PatternⅡb | Gender Male | -0.86 | 1.35 | -0.64 | 0.525 | 0.777 |
| PatternⅡc | (Intercept) | -2.87 | 0.52 | -5.51 | <0.001 | <0.001 |
| PatternⅡc | Country US | 0.36 | 0.38 | 0.94 | 0.346 | 0.599 |
| PatternⅡc | Country Japan | -0.06 | 0.35 | -0.17 | 0.862 | 1 |
| PatternⅡc | Dream Type (time) | -1.69 | 0.64 | -2.63 | 0.009 | 0.028 |
| PatternⅡc | Narrative Length (WC) | -0.003 | 0.004 | -0.65 | 0.515 | 0.768 |
| PatternⅡc | Age | 0.01 | 0.01 | 1.32 | 0.187 | 0.387 |
| PatternⅡc | Gender | -0.04 | 0.27 | -0.13 | 0.896 | 1 |
| PatternⅡc | Gender Female | 1.03 | 0.75 | 1.38 | 0.169 | 0.366 |
| PatternⅡc | Gender Male | 0.29 | 0.77 | 0.37 | 0.708 | 0.891 |
| PatternⅡd | (Intercept) | -1.36 | 0.40 | -3.42 | <0.001 | 0.003 |
| PatternⅡd | Country US | 1.10 | 0.31 | 3.55 | <0.001 | 0.002 |
| PatternⅡd | Country Japan | 0.85 | 0.30 | 2.83 | 0.005 | 0.016 |
| PatternⅡd | Dream Type (time) | -0.14 | 0.38 | -0.37 | 0.713 | 0.891 |
| PatternⅡd | Narrative Length (WC) | 0.009 | 0.002 | 4.04 | <0.001 | <0.001 |
| PatternⅡd | Age | -0.03 | 0.008 | -3.50 | <0.001 | 0.002 |
| PatternⅡd | Gender | 0.01 | 0.19 | 0.07 | 0.946 | 1 |
| PatternⅡd | Gender Female | -0.47 | 0.46 | -1.02 | 0.307 | 0.548 |
| PatternⅡd | Gender Male | -0.70 | 0.45 | -1.57 | 0.117 | 0.280 |
| PatternⅡ | (Intercept) | -0.70 | 0.31 | -2.23 | 0.026 | 0.074 |
| PatternⅡ | Country US | 0.87 | 0.24 | 3.64 | <0.001 | 0.001 |
| PatternⅡ | Country Japan | 0.75 | 0.22 | 3.34 | <0.001 | 0.003 |
| PatternⅡ | Dream Type (time) | -0.66 | 0.31 | -2.17 | 0.030 | 0.082 |
| PatternⅡ | Narrative Length (WC) | 0.006 | 0.002 | 3.19 | 0.001 | 0.005 |
| PatternⅡ | Age | -0.02 | 0.006 | -2.87 | 0.004 | 0.015 |
| PatternⅡ | Gender | -0.08 | 0.16 | -0.51 | 0.609 | 0.838 |
| PatternⅡ | Gender Female | -0.03 | 0.37 | -0.07 | 0.945 | 1 |
| PatternⅡ | Gender Male | -0.47 | 0.36 | -1.31 | 0.192 | 0.390 |
| PatternⅢa | (Intercept) | -3.20 | 0.94 | -3.39 | <0.001 | 0.003 |
| PatternⅢa | Country US | -2.09 | 0.96 | -2.17 | 0.030 | 0.082 |
| PatternⅢa | Country Japan | -2.22 | 0.84 | -2.63 | 0.008 | 0.028 |
| PatternⅢa | Dream Type (time) | -0.42 | 0.54 | -0.79 | 0.432 | 0.666 |
| PatternⅢa | Narrative Length (WC) | 0.001 | 0.01 | 0.08 | 0.937 | 1 |
| PatternⅢa | Age | 0.002 | 0.02 | 0.09 | 0.932 | 1 |
| PatternⅢa | Gender | 0.42 | 0.48 | 0.87 | 0.382 | 0.635 |
| PatternⅢa | Gender Female | -15.83 | 3363.72 | -0.005 | 0.996 | 1 |
| PatternⅢa | Gender Male | 0.04 | 1.30 | 0.03 | 0.973 | 1 |
| PatternⅢb | (Intercept) | -3.95 | 1.14 | -3.46 | <0.001 | 0.003 |
| PatternⅢb | Country US | -2.18 | 1.34 | -1.63 | 0.102 | 0.248 |
| PatternⅢb | Country Japan | -1.41 | 1.24 | -1.13 | 0.258 | 0.477 |
| PatternⅢb | Dream Type (time) | 1.12 | 0.82 | 1.36 | 0.173 | 0.367 |
| PatternⅢb | Narrative Length (WC) | <0.001 | 0.007 | 0.01 | 0.992 | 1 |
| PatternⅢb | Age | -0.02 | 0.022 | -0.86 | 0.392 | 0.639 |
| PatternⅢb | Gender | 0.41 | 0.68 | 0.60 | 0.548 | 0.800 |
| PatternⅢb | Gender Female | 1.05 | 1.46 | 0.72 | 0.471 | 0.719 |
| PatternⅢb | Gender Male | 1.84 | 1.39 | 1.33 | 0.184 | 0.387 |
| PatternⅢc | (Intercept) | -2.98 | 0.42 | -7.07 | <0.001 | <0.001 |
| PatternⅢc | Country US | -1.36 | 0.37 | -3.65 | <0.001 | 0.001 |
| PatternⅢc | Country Japan | -0.63 | 0.33 | -1.91 | 0.056 | 0.141 |
| PatternⅢc | Dream Type (time) | 0.84 | 0.31 | 2.74 | 0.006 | 0.021 |
| PatternⅢc | Narrative Length (WC) | 0.006 | 0.002 | 2.74 | 0.006 | 0.021 |
| PatternⅢc | Age | 0.02 | 0.008 | 2.48 | 0.013 | 0.040 |
| PatternⅢc | Gender | 0.07 | 0.24 | 0.30 | 0.765 | 0.923 |
| PatternⅢc | Gender Female | 0.37 | 0.43 | 0.86 | 0.387 | 0.639 |
| PatternⅢc | Gender Male | 0.22 | 0.42 | 0.53 | 0.598 | 0.838 |
| PatternⅢ | (Intercept) | -2.31 | 0.39 | -5.99 | <0.001 | <0.001 |
| PatternⅢ | Country US | -1.73 | 0.34 | -5.15 | <0.001 | <0.001 |
| PatternⅢ | Country Japan | -0.99 | 0.29 | -3.42 | <0.001 | 0.003 |
| PatternⅢ | Dream Type (time) | 0.68 | 0.26 | 2.56 | 0.011 | 0.033 |
| PatternⅢ | Narrative Length (WC) | 0.006 | 0.002 | 2.57 | 0.010 | 0.032 |
| PatternⅢ | Age | 0.01 | 0.008 | 1.98 | 0.048 | 0.124 |
| PatternⅢ | Gender | 0.18 | 0.22 | 0.85 | 0.397 | 0.639 |
| PatternⅢ | Gender Female | 0.55 | 0.39 | 1.40 | 0.162 | 0.360 |
| PatternⅢ | Gender Male | 0.56 | 0.38 | 1.50 | 0.134 | 0.315 |
| PatternⅣa | (Intercept) | -1.86 | 0.44 | -4.27 | <0.001 | <0.001 |
| PatternⅣa | Country US | -0.41 | 0.34 | -1.20 | 0.230 | 0.447 |
| PatternⅣa | Country Japan | 0.24 | 0.27 | 0.88 | 0.378 | 0.635 |
| PatternⅣa | Dream Type (time) | -1.39 | 0.44 | -3.14 | 0.002 | 0.006 |
| PatternⅣa | Narrative Length (WC) | -0.002 | 0.004 | -0.41 | 0.681 | 0.876 |
| PatternⅣa | Age | 0.004 | 0.009 | 0.42 | 0.674 | 0.872 |
| PatternⅣa | Gender | -0.12 | 0.22 | -0.52 | 0.603 | 0.838 |
| PatternⅣa | Gender Female | -0.16 | 0.61 | -0.26 | 0.797 | 0.951 |
| PatternⅣa | Gender Male | -0.13 | 0.57 | -0.22 | 0.823 | 0.967 |
| PatternⅣb | (Intercept) | -22.68 | 391.02 | -0.06 | 0.954 | 1 |
| PatternⅣb | Country US | 17.54 | 391.01 | 0.04 | 0.964 | 1 |
| PatternⅣb | Country Japan | 17.18 | 391.01 | 0.04 | 0.965 | 1 |
| PatternⅣb | Dream Type (time) | <0.001 | 214.84 | <0.001 | 1 | 1 |
| PatternⅣb | Narrative Length (WC) | <0.001 | 0.007 | -0.005 | 0.996 | 1 |
| PatternⅣb | Age | 0.01 | 0.03 | 0.38 | 0.705 | 0.891 |
| PatternⅣb | Gender | 1.30 | 0.84 | 1.54 | 0.124 | 0.293 |
| PatternⅣb | Gender Female | -0.33 | 214.84 | -0.002 | 0.999 | 1 |
| PatternⅣb | Gender Male | 0.93 | 214.84 | 0.004 | 0.997 | 1 |
| PatternⅣc | (Intercept) | -7.22 | 1.57 | -4.60 | <0.001 | <0.001 |
| PatternⅣc | Country US | 0.37 | 1.15 | 0.32 | 0.750 | 0.920 |
| PatternⅣc | Country Japan | -0.71 | 1.03 | -0.69 | 0.493 | 0.739 |
| PatternⅣc | Dream Type (time) | -18.03 | 5706.55 | -0.003 | 0.997 | 1 |
| PatternⅣc | Narrative Length (WC) | -0.02 | 0.02 | -0.85 | 0.396 | 0.639 |
| PatternⅣc | Age | 0.06 | 0.03 | 2.17 | 0.030 | 0.082 |
| PatternⅣc | Gender | 0.58 | 0.93 | 0.63 | 0.530 | 0.779 |
| PatternⅣc | Gender Female | 19.46 | 5706.55 | 0.003 | 0.997 | 1 |
| PatternⅣc | Gender Male | 0.90 | 7277.65 | <0.001 | 1.000 | 1 |
| PatternⅣd | (Intercept) | -22.43 | 0.004 | -5142.60 | 0 | 0 |
| PatternⅣd | Country US | 16.14 | 0.57 | 28.20 | <0.001 | <0.001 |
| PatternⅣd | Country Japan | 16.02 | 0.004 | 3674.20 | 0 | 0 |
| PatternⅣd | Dream Type (time) | -0.09 | 0.004 | -20.81 | <0.001 | <0.001 |
| PatternⅣd | Narrative Length (WC) | 0.007 | 0.006 | 1.14 | 0.255 | 0.477 |
| PatternⅣd | Age | -0.07 | 0.009 | -7.42 | <0.001 | <0.001 |
| PatternⅣd | Gender | 1.11 | 0.66 | 1.67 | 0.094 | 0.234 |
| PatternⅣd | Gender Female | 0.80 | 0.71 | 1.12 | 0.261 | 0.477 |
| PatternⅣd | Gender Male | 1.14 | 0.004 | 261.50 | 0 | 0 |
| PatternⅣe | (Intercept) | -5.16 | 1.33 | -3.89 | <0.001 | <0.001 |
| PatternⅣe | Country US | 1.25 | 1.07 | 1.16 | 0.244 | 0.463 |
| PatternⅣe | Country Japan | 0.96 | 1.09 | 0.88 | 0.380 | 0.635 |
| PatternⅣe | Dream Type (time) | 0.71 | 1.23 | 0.58 | 0.564 | 0.806 |
| PatternⅣe | Narrative Length (WC) | 0.01 | 0.003 | 4.62 | <0.001 | <0.001 |
| PatternⅣe | Age | 0.002 | 0.02 | 0.09 | 0.925 | 1 |
| PatternⅣe | Gender | -0.51 | 0.56 | -0.91 | 0.364 | 0.619 |
| PatternⅣe | Gender Female | -2.12 | 1.50 | -1.41 | 0.158 | 0.355 |
| PatternⅣe | Gender Male | -1.78 | 1.39 | -1.28 | 0.201 | 0.401 |
| PatternⅣf | (Intercept) | -44.73 | 0.006 | -7293.89 | 0 | 0 |
| PatternⅣf | Country US | 18.13 | 2.01 | 9.024 | <0.001 | <0.001 |
| PatternⅣf | Country Japan | 17.48 | 0.006 | 2850.83 | 0 | 0 |
| PatternⅣf | Dream Type (time) | -0.37 | 0.006 | -59.99 | 0 | 0 |
| PatternⅣf | Narrative Length (WC) | 0.008 | 0.005 | 1.42 | 0.156 | 0.354 |
| PatternⅣf | Age | -0.04 | 0.006 | -6.39 | <0.001 | <0.001 |
| PatternⅣf | Gender | 18.98 | 1.93 | 9.83 | <0.001 | <0.001 |
| PatternⅣf | Gender Female | -0.72 | 2048.35 | < -0.001 | 1.000 | 1 |
| PatternⅣf | Gender Male | 17.86 | 0.006 | 2912.50 | 0 | 0 |
| PatternⅣg | (Intercept) | -1.28 | 0.38 | -3.35 | <0.001 | 0.003 |
| PatternⅣg | Country US | 0.34 | 0.27 | 1.23 | 0.219 | 0.430 |
| PatternⅣg | Country Japan | -0.43 | 0.26 | -1.67 | 0.095 | 0.234 |
| PatternⅣg | Dream Type (time) | -0.38 | 0.29 | -1.30 | 0.193 | 0.390 |
| PatternⅣg | Narrative Length (WC) | -0.001 | 0.003 | -0.48 | 0.628 | 0.843 |
| PatternⅣg | Age | -0.004 | 0.008 | -0.44 | 0.657 | 0.859 |
| PatternⅣg | Gender | -0.10 | 0.20 | -0.50 | 0.617 | 0.838 |
| PatternⅣg | Gender Female | -1.01 | 0.45 | -2.25 | 0.024 | 0.071 |
| PatternⅣg | Gender Male | -0.19 | 0.37 | -0.51 | 0.611 | 0.838 |
| PatternⅣ | (Intercept) | -0.63 | 0.29 | -2.14 | 0.033 | 0.087 |
| PatternⅣ | Country US | 0.25 | 0.22 | 1.13 | 0.257 | 0.477 |
| PatternⅣ | Country Japan | 0.01 | 0.20 | 0.06 | 0.952 | 1 |
| PatternⅣ | Dream Type (time) | -0.86 | 0.25 | -3.44 | <0.001 | 0.003 |
| PatternⅣ | Narrative Length (WC) | 0.003 | 0.002 | 1.49 | 0.136 | 0.316 |
| PatternⅣ | Age | <0.001 | 0.006 | 0.15 | 0.880 | 1 |
| PatternⅣ | Gender | -0.01 | 0.15 | -0.07 | 0.942 | 1 |
| PatternⅣ | Gender Female | -0.39 | 0.34 | -1.17 | 0.243 | 0.463 |
| PatternⅣ | Gender Male | 0.02 | 0.31 | 0.07 | 0.945 | 1 |
| PatternⅤa | (Intercept) | -4.07 | 1.32 | -3.08 | 0.002 | 0.008 |
| PatternⅤa | Country US | 1.50 | 1.09 | 1.39 | 0.166 | 0.366 |
| PatternⅤa | Country Japan | 0.65 | 1.11 | 0.59 | 0.554 | 0.802 |
| PatternⅤa | Dream Type (time) | -15.36 | 200.82 | -0.08 | 0.939 | 1 |
| PatternⅤa | Narrative Length (WC) | 0.001 | 0.004 | 0.30 | 0.761 | 0.923 |
| PatternⅤa | Age | -0.03 | 0.02 | -1.30 | 0.193 | 0.390 |
| PatternⅤa | Gender | 0.01 | 0.65 | 0.01 | 0.988 | 1 |
| PatternⅤa | Gender Female | 15.50 | 200.82 | 0.08 | 0.938 | 1 |
| PatternⅤa | Gender Male | 15.99 | 200.82 | 0.08 | 0.937 | 1 |
| PatternⅤb | (Intercept) | -24.62 | 0.004 | -6277.38 | 0 | 0 |
| PatternⅤb | Country US | 16.23 | 0.92 | 17.63 | <0.001 | <0.001 |
| PatternⅤb | Country Japan | 14.35 | 1.29 | 11.17 | <0.001 | <0.001 |
| PatternⅤb | Dream Type (time) | 17.49 | 0.004 | 4461.05 | 0 | 0 |
| PatternⅤb | Narrative Length (WC) | 0.007 | 0.003 | 2.05 | 0.040 | 0.106 |
| PatternⅤb | Age | -0.005 | 0.004 | -1.37 | 0.169 | 0.366 |
| PatternⅤb | Gender | -18.11 | 0.004 | -4617.77 | 0 | 0 |
| PatternⅤb | Gender Female | -16.75 | 1.28 | -13.08 | <0.001 | <0.001 |
| PatternⅤb | Gender Male | -17.41 | 0.96 | -18.05 | <0.001 | <0.001 |
| PatternⅤc | (Intercept) | -2.41 | 0.46 | -5.22 | <0.001 | <0.001 |
| PatternⅤc | Country US | 0.67 | 0.34 | 1.98 | 0.048 | 0.124 |
| PatternⅤc | Country Japan | -0.85 | 0.35 | -2.41 | 0.016 | 0.049 |
| PatternⅤc | Dream Type (time) | 0.13 | 0.36 | 0.35 | 0.724 | 0.893 |
| PatternⅤc | Narrative Length (WC) | -0.002 | 0.003 | -0.82 | 0.411 | 0.652 |
| PatternⅤc | Age | 0.002 | 0.009 | 0.22 | 0.825 | 0.967 |
| PatternⅤc | Gender | -0.15 | 0.26 | -0.59 | 0.557 | 0.802 |
| PatternⅤc | Gender Female | 0.42 | 0.46 | 0.91 | 0.363 | 0.619 |
| PatternⅤc | Gender Male | 0.65 | 0.44 | 1.48 | 0.139 | 0.320 |
| PatternⅤd | (Intercept) | -2.70 | 0.66 | -4.07 | <0.001 | <0.001 |
| PatternⅤd | Country US | 0.06 | 0.52 | 0.11 | 0.914 | 1 |
| PatternⅤd | Country Japan | -0.50 | 0.53 | -0.94 | 0.347 | 0.599 |
| PatternⅤd | Dream Type (time) | 0.30 | 0.55 | 0.55 | 0.583 | 0.823 |
| PatternⅤd | Narrative Length (WC) | 0.003 | 0.003 | 0.95 | 0.341 | 0.599 |
| PatternⅤd | Age | -0.01 | 0.01 | -1.10 | 0.269 | 0.489 |
| PatternⅤd | Gender | -0.22 | 0.39 | -0.56 | 0.575 | 0.817 |
| PatternⅤd | Gender Female | 0.26 | 0.70 | 0.37 | 0.713 | 0.891 |
| PatternⅤd | Gender Male | 0.56 | 0.67 | 0.84 | 0.401 | 0.642 |
| PatternⅤe | (Intercept) | -2.16 | 0.42 | -5.11 | <0.001 | <0.001 |
| PatternⅤe | Country US | -1.55 | 0.37 | -4.18 | <0.001 | <0.001 |
| PatternⅤe | Country Japan | -0.59 | 0.32 | -1.85 | 0.065 | 0.162 |
| PatternⅤe | Dream Type (time) | 0.89 | 0.30 | 2.96 | 0.003 | 0.011 |
| PatternⅤe | Narrative Length (WC) | 0.008 | 0.002 | 3.34 | <0.001 | 0.003 |
| PatternⅤe | Age | <0.001 | 0.008 | 0.02 | 0.981 | 1 |
| PatternⅤe | Gender | -0.06 | 0.24 | -0.24 | 0.813 | 0.965 |
| PatternⅤe | Gender Female | 0.30 | 0.42 | 0.71 | 0.477 | 0.721 |
| PatternⅤe | Gender Male | 0.48 | 0.40 | 1.18 | 0.236 | 0.456 |
| PatternⅤ | (Intercept) | -1.09 | 0.32 | -3.43 | <0.001 | 0.003 |
| PatternⅤ | Country US | -0.13 | 0.25 | -0.50 | 0.615 | 0.838 |
| PatternⅤ | Country Japan | -0.52 | 0.24 | -2.20 | 0.028 | 0.079 |
| PatternⅤ | Dream Type (time) | 0.78 | 0.24 | 3.20 | 0.001 | 0.005 |
| PatternⅤ | Narrative Length (WC) | 0.005 | 0.002 | 2.57 | 0.010 | 0.032 |
| PatternⅤ | Age | -0.003 | 0.006 | -0.45 | 0.653 | 0.859 |
| PatternⅤ | Gender | -0.19 | 0.18 | -1.05 | 0.293 | 0.527 |
| PatternⅤ | Gender Female | 0.15 | 0.32 | 0.48 | 0.628 | 0.84 |
| PatternⅤ | Gender Male | 0.30 | 0.30 | 0.99 | 0.323 | 0.572 |
