# Supplementary material for: Examining the association between cultural self-construal and dream structures in China, Japan, and the United States
Source: Front Psychol. 2026 Jan 12;16:1688407. doi: 10.3389/fpsyg.2025.1688407 (PMC12832506; doi:10.3389/fpsyg.2025.1688407)
Supplement: Supplementary file 4 [file Table_2.docx]

**Supplementary Material 2: Fit indices for the multigroup CFA models (configural, metric, and scalar) across the four scales**

**Supplementary Table S2**

| **Scale** | **Model** | **CFI** | **TLI** | **RMSEA** | **SRMR** |
| --- | --- | --- | --- | --- | --- |
| Anthropophobia mentality | Configural | 0.766 | 0.748 | 0.130 | 0.069 |
| Anthropophobia mentality | Metric | 0.761 | 0.755 | 0.128 | 0.092 |
| Anthropophobia mentality | Scalar | 0.714 | 0.720 | 0.137 | 0.104 |
| Sense of Self | Configural | 0.613 | 0.574 | 0.122 | 0.127 |
| Sense of Self | Metric | 0.591 | 0.577 | 0.122 | 0.146 |
| Sense of Self | Scalar | 0.504 | 0.516 | 0.130 | 0.155 |
| Independent view of self | Configural | 0.869 | 0.831 | 0.093 | 0.059 |
| Independent view of self | Metric | 0.853 | 0.839 | 0.091 | 0.078 |
| Independent view of self | Scalar | 0.771 | 0.780 | 0.106 | 0.095 |
| Interdependent view of self | Configural | 0.746 | 0.674 | 0.111 | 0.079 |
| Interdependent view of self | Metric | 0.718 | 0.691 | 0.108 | 0.093 |
| Interdependent view of self | Scalar | 0.497 | 0.518 | 0.134 | 0.120 |
