# Supplementary material for: Examining the association between cultural self-construal and dream structures in China, Japan, and the United States
Source: Front Psychol. 2026 Jan 12;16:1688407. doi: 10.3389/fpsyg.2025.1688407 (PMC12832506; doi:10.3389/fpsyg.2025.1688407)
Supplement: Supplementary file 5 [file Table_3.docx]

**Supplementary Material 3: Pattern V Subcategory Summary Tables**

| **Supplementary Table 3.** Pattern V summary for “impressive dreams in childhood” | | | |
| --- | --- | --- | --- |
| Subclassification of Pattern V | Chinese（％） | American（％） | Japanese（％） |
| “The dream-ego wants to get into contact but is ignored by others” | 0.6 | 1.4 | 2.3 |
| “The dream-ego is criticized, devalued or made ridiculous by others and feels shame” | 0.0 | 1.4 | 0.0 |
| “The dream-ego is successful in creating the desired contact” | 9.8 | 14.1 | 6.6 |
| “A special case: the dream ego is aggressive towards others (even kills others) which expresses the will of the dream ego to be separated and autonomous” | 3.5 | 5.5 | 1.2 |
| “A special case: Positive behavior of others to the dream-ego” | 11.6 | 5.0 | 6.6 |
| Note: The table presents the rate% in parenthesis for each situation in Chinese (n = 173), American (n = 220) and Japanese (n = 257) samples. For example, the rate of the situation that “The dream-ego wants to get into contact but is ignored by others” in Chinese samples is calculated by dividing the number of dreams with the situation that “The dream-ego wants to get into contact but is ignored by others” by the total number of dreams in the Chinese samples. | | | |

| **Supplementary Table 4.** Pattern V summary for “recent impressive dreams” | | | |
| --- | --- | --- | --- |
| Subclassification of Pattern V | Chinese（％） | American（％） | Japanese（％） |
| “The dream-ego wants to get into contact but is ignored by others” | 0.0 | 5.9 | 0.4 |
| “The dream-ego is criticized, devalued or made ridiculous by others and feels shame” | 1.2 | 2.3 | 0.8 |
| “The dream-ego is successful in creating the desired contact” | 11.0 | 30.0 | 7.4 |
| “A special case: the dream ego is aggressive towards others (even kills others) which expresses the will of the dream ego to be separated and autonomous” | 4.6 | 8.2 | 5.8 |
| “A special case: Positive behavior of others to the dream-ego” | 23.7 | 12.3 | 23.3 |
| Note: The table presents the rate% in parenthesis for each situation in Chinese (n = 173), American (n = 220) and Japanese (n = 257) samples. For example, the rate of the situation that “The dream-ego wants to get into contact but is ignored by others” in Chinese samples is calculated by dividing the number of dreams with the situation that “The dream-ego wants to get into contact but is ignored by others” by the total number of dreams in the Chinese samples. | | | |
